# Electrochemically activated spinel manganese oxide for rechargeable aqueous aluminum battery

**DOI:** 10.1038/s41467-018-07980-7

**Published:** 2019-01-08

**Authors:** Chuan Wu, Sichen Gu, Qinghua Zhang, Ying Bai, Matthew Li, Yifei Yuan, Huali Wang, Xinyu Liu, Yanxia Yuan, Na Zhu, Feng Wu, Hong Li, Lin Gu, Jun Lu

**Affiliations:** 1 School of Materials Science and Engineering, Beijing Institute of Technology, No. 5 South Zhongguancun Street, Beijing, 100081 China; 2 Collaborative Innovation Center of Electric Vehicles in Beijing, No. 5 South Zhongguancun Street, Beijing, 100081 China; 3 Institute of Physics, Chinese Academy of Sciences, No. 8 Zhongguancun South 3rd Street, Beijing, 100190 China; 4 Chemical Sciences and Engineering Division, Argonne National Laboratory, Lemont, IL 60439 USA

## Abstract

Aluminum is a naturally abundant, trivalent charge carrier with high theoretical specific capacity and volumetric energy density, rendering aluminum-ion batteries a technology of choice for future large-scale energy storage. However, the frequent collapse of the host structure of the cathode materials and sluggish kinetics of aluminum ion diffusion have thus far hampered the realization of practical battery devices. Here, we synthesize Al_*x*_MnO_2_·*n*H_2_O by an in-situ electrochemical transformation reaction to be used as a cathode material for an aluminum-ion battery with a configuration of Al/Al(OTF)_3_-H_2_O/Al_*x*_MnO_2_·*n*H_2_O. This cell is not only based on aqueous electrolyte chemistry but also delivers a high specific capacity of 467 mAh g^−1^ and a record high energy density of 481 Wh kg^−1^. The high safety of aqueous electrolyte, facile cell assembly and the low cost of materials suggest that this aqueous aluminum-ion battery holds promise for large-scale energy applications.

## Abstract (web summary)

The instability of the host structure of cathode materials and sluggish aluminium ion diffusion are the major challenges facing the Al-ion battery. Here the authors show Al_*x*_MnO_2_·*n*H_2_O as a cathode that allows for reversible Al^3+^ (de)intercalation in an aqueous electrolyte and impressive electrochemical performance for a battery device.

## Introduction

The development of renewable energy resources, such as solar and wind power, calls for the corresponding large-scale energy storage system^1^. Being widely employed in portable electronics^2^, lithium-ion batteries are among the most successful energy storage systems^1,3^. However, the limited nature of lithium supply makes it challenging to sustainably satisfy all the urgent demands for grid-scale energy storage systems and other applications^1,3,4^. Rechargeable Al batteries emerge as a competitive alternative for post-lithium batteries^5,6^. As typical multi-electron reaction devices^5,7^, the Al-ion batteries possess the potential of higher specific capacity, superior volumetric energy density, and comparable gravimetric energy density to lithium-ion batteries^6,8,9^. Moreover, the high abundancy and easy accessibility of Al resources enable Al-ion batteries to become an ideal candidate for large-scale energy storage system^9^.

Because the standard electrode potential of Al^3+^/Al (−1.68 V) is lower than H^+^/H_2_, the evolution of H_2_ occurs due to the reaction between aluminum foil and aqueous acid or alkali solution. Thus, Al cannot be electrochemically striped or deposited in a common aqueous solution. To be compatible with Al anode, the ionic liquid AlCl_3_/[EMIM]Cl with a wider range of electrochemical active window emerges as the typical electrolyte, which provides a mild corrosive effect on the Al surface to activate the Al striping and plating reaction. However, such type of ionic liquid electrolytes are not preferable for the application in large-scale energy storage systems due to its high cost and potential environmental concerns. Therefore, an alternative non-flammable and low-toxicity aqueous electrolytes for low-cost rechargeable aluminum-ion battery is urgently needed^10,11^.

Another critical issue that limits the application of Al batteries is the low energy density due to the lack of proper cathode materials. Thus far, there are two categories of cathode materials for rechargeable Al batteries. One is the carbon-based materials with high specific surface area such as 3D graphite-foam that can accommodate Al_*x*_Cl_*y*_^−^^12–18^. Owing to the ultrafast monovalent reaction kinetics^18^, the 3D graphite-foam delivers a high power density of 3000 W kg^−1^ ^12^. At the same time, the monovalent reaction inherently limits the obtainable specific capacity. Among the carbon-based materials, the highest reported specific capacity (graphene nanoribbons on highly porous 3D-graphene foam) was only 148 mAh g^−1^ ^13^^,^ which is far from practical requirements. The other category of cathode materials can realize trivalent reaction and thus have the potential to achieve high specific capacity, but they suffer from relatively lower redox potentials. It is well known that the strong electrostatic nature of Al^3+^ always leads to sluggish kinetics^8,19^, high over-potentials^8,19^, and the eventual collapse of host structure^19,20^. Therefore, to accommodate trivalent Al^3+^, it is essential for the cathode materials to possess weak bond strengths between the host frameworks (namely, moderate polarity). The representatives with moderate polarity are sulfur^19,21^, transition metal sulfides^22–26^, Prussian blue analogues (PBAs)^10,27,28^, and some transition metal oxide (transition metal = V^7,29–31^ or Ti^11,32^). These materials have promoted a relatively reversible trivalent reaction, but with discharge voltages only ranging from 0.3 to 0.8 V can hardly be considered as valid cathode materials. As such, there is an urgent need for the development of cathode materials for Al batteries with high capacity and high redox potential.

Herein, an aqueous rechargeable aluminum-ion battery in the form of Al/Al(OTF)_3_-H_2_O/Al_*x*_MnO_2_·*n*H_2_O is proposed. This battery chemistry not only realizes reversible ex/insertion of Al^3+^ in an aqueous electrolyte, but also for the first time, successfully accomplishes the trivalent reaction at a high redox potential. The Al_*x*_MnO_2_·*n*H_2_O as a cathode is synthesized by introducing H_2_O and Al^3+^ into spinel Mn_3_O_4_ in the trivalent cation (Al^3+^) aqueous solution via an in-situ electrochemical transformation reaction. The cathode material Al_*x*_MnO_2_·*n*H_2_O imposes a high operation voltage to accommodate Al^3+^ ions(1.2 V). In addition, the aqueous electrolyte and crystal water in Al_*x*_MnO_2_·*n*H_2_O molecule shield the electrostatic interaction between Al^3+^ and host frameworks, which enables reversible trivalent reactions. With the aqueous electrolyte Al(OTF)_3_-H_2_O, the Al_*x*_MnO_2_·*n*H_2_O cathode exhibits a reversible discharge capacity of 467 mAh g^−1^ with the discharge plateau of 1.2 V. To the best of our knowledge, the present specific capacity and plateau are among the highest values reported for the trivalent reaction cathodes and all the aqueous rechargeable aluminum-ion batteries^33^.

## Results

### Preparation and characterization of Al_*x*_MnO_2_·*n*H_2_O

The aqueous aluminum trifluoromethanesulfonate (Al(OTF)_3_) (5 mol L^−1^) solution was used as the electrolyte. The overall stable window for this aqueous electrolyte was determined as the voltage range of −0.3 to 3.3 V (vs. Al/Al^3+^) by the method of cyclic voltammetry (CV) as shown in Supplementary Figure 1a. Accordingly, the electrochemical transformation process was conducted in the voltage range of 0.5–1.8 V. Within this voltage range, both the Al(OTF)_3_ electrolyte is stabilized and the Al can be striped and plated on the anode side, while Al^3+^ can be inserted and extracted from the cathode materials without triggering any side reaction.

Inspired by the reports of spinel-to-layered reaction^34–36^, we design an electrochemical reaction and aim to transform spinel Mn_3_O_4_ into layered A_*x*_MnO_2_·*n*H_2_O in aqueous solution. In detail, Mn_3_O_4_ nanoparticles were used as precursors and assembled into a cell with the electrolyte of Al(OTF)_3_-H_2_O (5 mol L^−1^) and anode of Al foil (Al/Al(OTF)_3_-H_2_O/Mn_3_O_4_) in order to obtain Al_*x*_MnO_2_·*n*H_2_O. The cell was charged to 1.8 V (vs. Al/Al^3+^) with a current density of 30 mAh g^−1^, and the corresponding charging profile is shown in Supplementary Figure 2c.

**Fig. 1:**
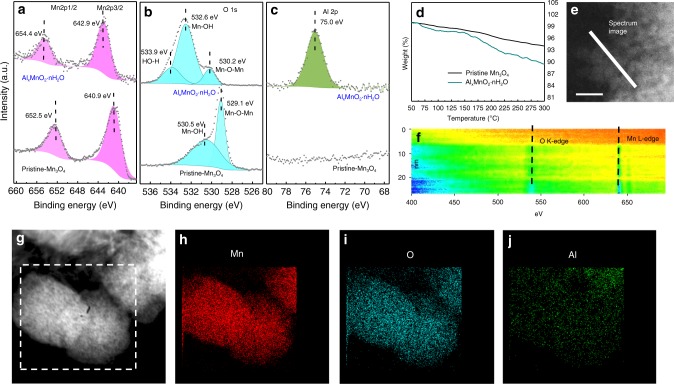
The evidences of electrochemical transformation from spinel Mn_3_O_4_ into layered A_*x*_MnO_2_·*n*H_2_O. **a** Mn2p, **b** O1s XPS, and **c** Al2p spectra of Mn_3_O_4_ and the Al_*x*_MnO_2_·*n*H_2_O. **d** DTG curve for Mn_3_O_4_ and Al_*x*_MnO_2_·*n*H_2_O. **e** HAADF image for EELS line scanning pathway as indicated by the white line. **f** Mn L-edge and O K-edge EELS spectrum along the scanning pathway in **e**. **g** Low-magnification STEM image and **h**–**j** element mappings of a Al_*x*_MnO_2_·*n*H_2_O: **h** Mn, **i** O, **j** Al. Scale bar: 10 nm for **e**

To identify the products prepared by the electrochemical transformation, electron energy loss spectroscopy (EELS), X-ray photoelectron spectroscopy (XPS), thermogravimetric analysis (TGA), and transmission electron microscope-energy dispersive X-ray spectroscopy (TEM-EDS) were carried out to investigate the element identities and valence state of the products. As seen in Fig. 1a–c, the XPS spectra demonstrate the variation of chemical valance state after electrochemical transformation. In the pristine Mn_3_O_4_, the Mn2p3/2 and Mn2p1/2 observed at 640.9 and 652.5 eV are characteristics of Mn (II/III) in Mn_3_O_4_. After electrochemical transformation, Mn2p3/2 and Mn2p1/2 shift higher to 642.9 and 654.4 eV, which is in accordance with the Mn(IV) in MnO_2_^37^. As for the O1s, a shoulder peak appears at 533.9 eV, which can be assigned to H–OH^38,39^. The binding energy of 75.0 eV is attributed to Al^3+^ that engaged into the host materials. The variation of valance states of Mn and O are further confirmed from the slight intensity variation of oxygen K-edge and manganese L-edge over the thin edge of the sample (Fig. 1e, f). The involvement of crystal water in the framework of materials is proved by the TGA in Fig. 1d. Compared with precursor Mn_3_O_4_, the product shows a more significant weight loss in the range of 50–300 °C, resulted from crystal water loss. The TEM-EDS in Fig. 1g–j provides the information on the distribution of Al. Thus, all the results suggest that the products are Al containing manganese dioxide with crystal water, verifying the electrochemical transformation of Mn_3_O_4_ → Al_*x*_MnO_2_·*n*H_2_O.

**Fig. 2:**
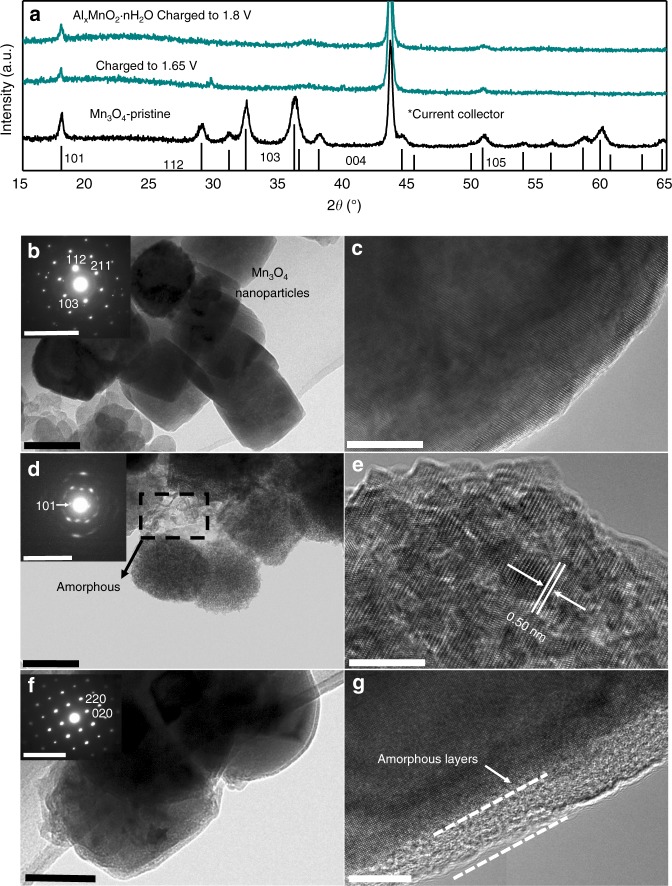
Structure characterization of Al_*x*_MnO_2_·*n*H_2_O. **a** XRD patterns of Mn_3_O_4_ and Al_*x*_MnO_2_·*n*H_2_O. **b**, **c** TEM image of the pristine Mn_3_O_4_, inset: SAED spectra. **d**, **e** TEM image of Al_*x*_MnO_2_·*n*H_2_O, inset: SAED spectra. **f**, **g** TEM image of Mn_3_O_4_ after discharge, inset: SAED spectra. Scale bar: 100 nm for **b**, **d**, **f**; 10 nm for **c**, **e**, **g**; 10 1/nm for inset in **b**, **d**, **f**

To further probe the structure of the as-prepared Al_*x*_MnO_2_·*n*H_2_O, X-ray diffraction (XRD) and TEM were conducted. As shown by XRD and TEM image in Fig. 2a–c, the precursor is nanoparticle with well-faceted surfaces, and its XRD and selected area electron diffraction (SAED, Fig. 2b inset) match well with the pattern of pure spinel Mn_3_O_4_ (JCPDS File No. #24-0734). After electrochemical transformation, all the sharp peaks of Mn_3_O_4_ vanish except the (101) peak at 18°, indicating pristine spinel phase gradually transforms to the amorphous phase. The morphology of the nanoparticles is mostly preserved (seen in Fig. 2d, e), with slight coarseness emerging and peeling of amorphous layers off the surface of the particles. The elongated spots in SAED in Fig. 2d inset display the ring shapes, confirming the emergence of the amorphous phase again. Meanwhile, the residual peak at 18° in XRD spectrum infers layered structure materials with *d* spacing of 4.94 Å, which is in accordance with the observation of the exposed (101) point in SAED spectra in Fig. 2d. Furthermore, high-resolution transmission electron microscopy (HRTEM) spectra in Fig. 2e shows distinct layered lattice on the edges of the Al_*x*_MnO_2_·*n*H_2_O particles. Tentatively, it could be speculated that on galvanostatic charging to 1.8 V, the spinel Mn_3_O_4_ transforms into Al_*x*_MnO_2_·*n*H_2_O with a mixed phase of amorphous and layered structure. Such spinel to layer transition only takes place in charging process, evidenced by the direct discharge products of Mn_3_O_4_ nanoparticles in the same electrolyte as shown in Fig. 2f, g, which contains only amorphous layers on the surface of materials without layered structure.

**Fig. 3:**
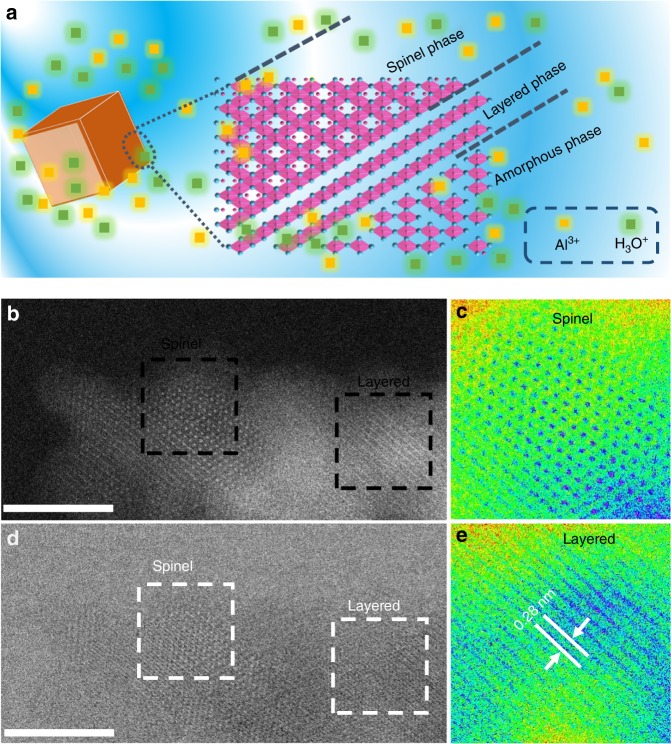
Schematic profile of the mix phase structure of Al_*x*_MnO_2_·*n*H_2_O. **a** The schematic profile of the structure of Al_*x*_MnO_2_·*n*H_2_O. **b** STEM-HAADF image of a thin edge of Al_*x*_MnO_2_·*n*H_2_O. **c** A false-colored image of the spinel structure areas quoted by white dash line in **d**. **d** STEM-ABF image. **e** A false-colored image of the layered structure areas quoted by white dash line in **d**. Scale bar: 5 nm for **b**, **d**

To identify the detailed mixed phase, scanning transmission electron microscopy (STEM), Z-contrast STEM-high angle annular dark field (STEM-HAADF), and annular bright-field (ABF) on the thin edge region of the Al_*x*_MnO_2_·*n*H_2_O particles were employed. As shown in Fig. 3b–d, the atomic arrays marked by rectangle clearly indicate spinel and layered phase, respectively, confirming the structure of Al_*x*_MnO_2_·*n*H_2_O. Together with the evolution of valence state, it is reasonable to conclude that galvanostatic charging in Al(OTF)_3_-H_2_O solution enables the transition of spinel Mn_3_O_4_ to a mixed phase of the layer and amorphous structure of Al_*x*_MnO_2_·*n*H_2_O. It should be noted that the STEM (Fig. 3b–e) image shows some scattered spinel phase retains after electrochemical transformation, but the amount is so little that it cannot be detected by XRD (Fig. 2a).

As we know, the structure transition (of cathode Mn containing material) from layered to spinel originates from the preferred migration of Mn which usually leads to capacity reduction^34,35,40^. Hence, the reverse process from spinel to layered structure seems quite surprising, because it is in the opposite direction to the known spontaneous processes^35^. However, this unconventional behavior of Mn_3_O_4_ has been elucidated in the electrochemical cycling process with an aqueous solution of Li, Na, and Mg^34,36^. In that typical process, the Mn^2+^ in the tetrahedral sites and some Mn^3+^ in octahedral sites dissolve out, while the other Mn^3+^ in the octahedral sites are oxidized to Mn^4+^. With the dissolving Mn rearrange into layered birnessite phase^35,41^, strains and defects are induced at the phase boundaries between the layered phase and the original spinel phase, where the water molecular and charge carriers insert into^34–36^. As a result, layered birnessite structure A_*x*_MnO_2_·*n*H_2_O (A = monovalent or divalent metal) with the *d* spacing of 7.1 Å and lamellar morphology are generated^34–36^. This spinel-to-layered process is a complicated formation process of new phase involving structure rearrangement and evolution of valance states^36^, where the water molecular plays an indispensable role.

Based on the knowledge as mentioned above, the structural evolution of the Mn_3_O_4_ in aqueous Al(OTF)_3_ is similar to the spinel-to-layered process of Li, Na, Mg in aqueous solution, but not exactly the same, because of the marked difference between the mixed phase and birnessite structure. Since the spinel-to-layered transformation process is determined by the efficiency of cations species in the aqueous electrolyte^36^, the formation of this mix phase is a result from the trivalent effect of Al^3+^ in the Al(OTF)_3_-H_2_O solution. To further understand the trivalent effects on the formation of Al_*x*_MnO_2_·*n*H_2_O on the atomic level, the crucial transformation steps were investigated based on first-principles calculations. The spin-to-layered transition process is initiated by dissolution of Mn^2+^ in the tetrahedral site which leaves the chemical composition of the spinel compound as Mn_2_O_4_^42^ (Supplementary Figure 3). Then the crucial step occurs as the water molecular and charge carriers compete to intercalate into the vacant site to from molecular inserted phase AMn_2_O_4_ (A = H_2_O, Li^+^, Na^+^, and Al^3+^). To investigate the thermodynamic driving force of this insertion step, the energy difference between the spinel Mn_2_O_4_ and AMn_2_O_4_ were compared in Supplementary Figure 3. The Al_*x*_Mn_2_O_4_ is the most thermodynamically avored species, implying the difficulty of insertion of the H_2_O and the failure of formation of birnessite phase in Al(OTF)_3_-H_2_O solution. Besides, owing to the lower formation energy, the Al^3+^ is much more likely to intercalate into the vacant site than Na^+^ and Li^+^, which rationalize the difference in the structure of the Al_*x*_MnO_2_·*n*H_2_O and birnessite phase formed in Na^+^, Li^+^ aqueous solution.

Accordingly, the hypothetical mechanism for the formation of Al_*x*_MnO_2_·*n*H_2_O is illustrated in Fig. 3a and Supplementary Figure 4. Since the layered structures with the *d* spacing of 4.95 and 2.8 Å were observed from different directions in Figs. 2e and 3b–e, it can be speculated that the layered phase in Al(OTF)_3_-H_2_O solution is produced by the dissolution of Mn in the tetrahedral site and some of octahedral sites. The dissolved Mn produced amorphous structures along the layered phase (Figs. 2e and 3c, e) in Al(OTF)_3_-H_2_O, instead of forming birnessite phase in aqueous solution of Li, Na, and Mg^34–36^. The influence of Al^3+^ concentration on the morphology and yields of Al_*x*_MnO_2_·*n*H_2_O are further discussed in Supplementary Figure 5 and Supplementary Figure 6.

This is the first time for spinel-to-layered process reported in the trivalent ions aqueous solution. In stark contrast from the birnessite structure in mono or bivalent ions aqueous solution, this Al_*x*_MnO_2_·*n*H_2_O shows its unique mixed phase of layered and amorphous structure in the trivalent ions aqueous solution.

### Electrochemical performance

**Fig. 4:**
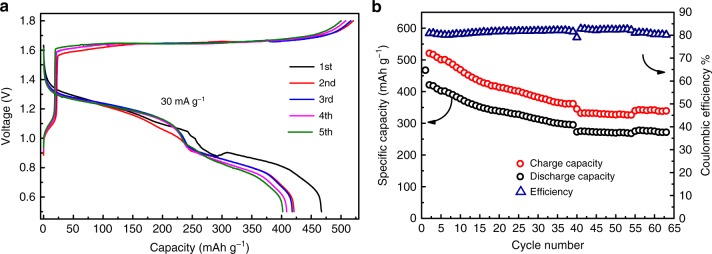
Electrochemical performance of Al/Al(OTF)_3_-H_2_O/Al_*x*_MnO_2_·*n*H_2_O rechargeable battery. **a** Galvanostatic charge and discharge profile. **b** Efficiency and cycling ability

To evaluate the electrochemical performance of Al_*x*_MnO_2_·*n*H_2_O, Al/Al(OTF)_3_-H_2_O/Al_*x*_MnO_2_·*n*H_2_O rechargeable aluminum-ion battery was assembled. Figure 4a, b shows the galvanostatic charge and discharge profiles together with the corresponding capacity retention. In the initial charge process, the Al_*x*_MnO_2_·*n*H_2_O electrode shows a short plateau at ca. 1.3 V and a long plateau at ca. 1.65 V, corresponding to Al ion extraction from the cathode materials accompanied by the oxidation of manganese. The 1st discharge capacity is as high as 467 mAh g^−1^, which is among the highest specific capacities achieved by rechargeable aluminum batteries (see the comparison of its electrochemical performance with reported results in Supplementary Table 2). Moreover, the discharge plateaus of Al_*x*_MnO_2_·*n*H_2_O are 1.2 and 0.8 V, with an average potential (1.1 V) which is superior to all other reported trivalent electron reactions cathodes^19,20,22–24^. Benefiting from the high specific capacity and average potential, the Al_*x*_MnO_2_·*n*H_2_O electrode affords an outstanding energy density of 481 Wh kg^−1^.

Other cathode materials for rechargeable aluminum-ion batteries, such as sulfur and metal sulfide materials, exhibit higher initial discharge capacity^19,26,43^. However, the dramatic capacity decay is a still of much concern. By contrast, the Al_*x*_MnO_2_·*n*H_2_O not only delivers high specific capacity, but also shows good cycling stability. Even after 60 cycles, its discharge capacity retains 272 mAh g^−1^ with the discharge plateau of 1.2 V. All in all, to the best of our knowledge, Al/Al(OTF)_3_-H_2_O/Al_*x*_MnO_2_·*n*H_2_O battery exhibits satisfactory comprehensive electrochemical performance that is far superior to other batteries with trivalent reaction cathode materials.

### Confirmation of the trivalent mechanism

**Fig. 5:**
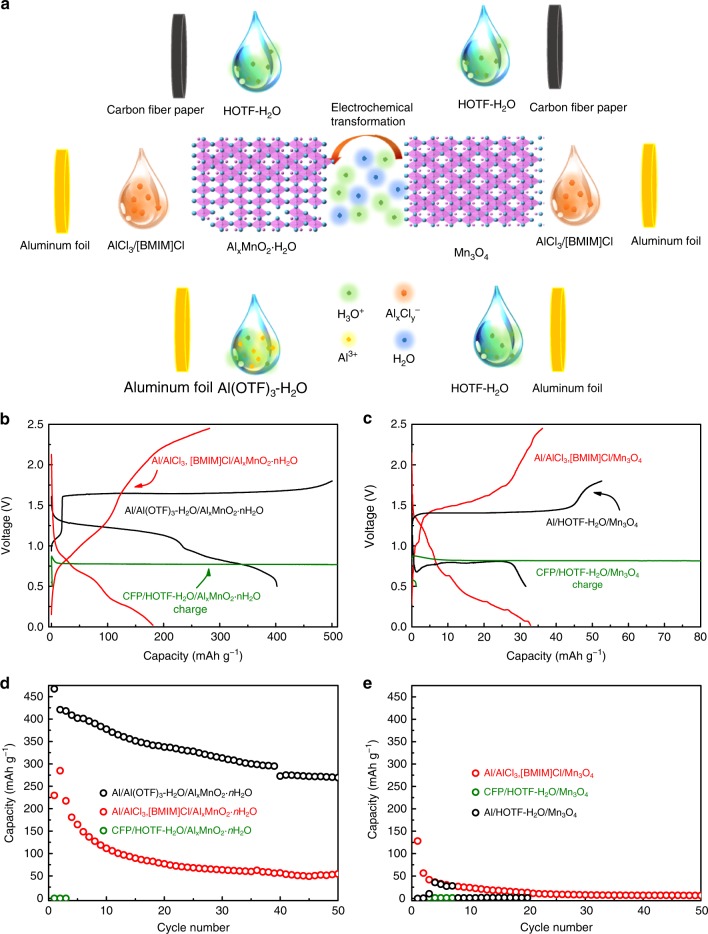
The schematic diagram and results of comparative experiment. **a** The scheme of the control experiment, it displays the experiment design that the cathode Al_*x*_MnO_2_·*n*H_2_O and Mn_3_O_4_ matches up with aqueous and ionic liquid electrolyte and counter anode respectively. **b**, **d** Typical charge and discharge of the different battery. **c**, **e** Discharge capacity of different battery

As inferred, the superior capacity and stability depends on the trivalent reaction and the aqueous electrolyte in Al/Al(OTF)_3_-H_2_O/Al_*x*_MnO_2_·*n*H_2_O. A series of comparative experiments using spinel Mn_3_O_4_ or Al_*x*_MnO_2_·*n*H_2_O as the cathodes with three kinds o`f electrolytes and paired with different anode were conducted to further understand the trivalent reaction and the role of the aqueous electrolyte in electrochemical performance. The aqueous HOTF electrolyte is used to identify the Al^3+^ contribution in capacity, the ionic liquid AlCl_3_/[BMIM]Cl is used as comparison to demonstrate the influence of the aqueous electrolyte on electrochemical performance as well. The ion species in the comparative samples are listed in Table 1 and the experiment diagram and results are shown in Fig. 5.

**Table 1:** The ion species and solvent molecular contained in the electrolyte of comparative samples

Coin cell	Electrolyte	Ion species in electrolyte
Al/Al(OTF)_3_-H_2_O/Al_*x*_MnO_2_·*n*H_2_O	Aqueous	Al^3+^ H_3_O^+^ OTF^−^
CFP/HOTF-H_2_O/Al_*x*_MnO_2_·*n*H_2_O	Aqueous	H_3_O^+^ OTF^−^
CFP/HOTF-H_2_O/Mn_3_O_4_	Aqueous	H_3_O^+^ OTF^−^
Al/HOTF-H_2_O/Mn_3_O_4_	Aqueous	Al^3+^ H_3_O^+^ OTF^−^
Al/AlCl_3_, [BMIM]Cl/Al_*x*_MnO_2_·*n*H_2_O	Ion liquid (non-aqueous)	Al_*x*_Cl_*y*_^−^ BMIM^+^
Al/AlCl_3_, [BMIM]Cl/Mn_3_O_4_	Ion liquid (non-aqueous)	Al_*x*_Cl_*y*_^−^ BMIM^+^

In addition to Al^3+^, the H_3_O^+^ with concentration of 3.16 mol L^−1^ is presented in Al(OTF)_3_-H_2_O (Supplementary Figure 2a, Table 1), both the Al^3+^ and H_3_O^+^ may contribute to the capacities. To make sure the capacity of Al/Al(OTF)_3_-H_2_O/Al_*x*_MnO_2_·*n*H_2_O is the result of trivalent Al^3+^ intercalation, CFP/HOTF-H_2_O/Mn_3_O_4_ and CFP/HOTF-H_2_O/Al_*x*_MnO_2_·*n*H_2_O coin cells without Al^3+^ in electrolyte were assembled and investigated. The concentration of H_3_O^+^ in HOTF-H_2_O (3.16 mol L^−1^) is consistent with that of Al(OTF)_3_-H_2_O (5 mol L^−1^) (Supplementary Figure 2a, b). In those two cells, neither the HOTF-H_2_O nor CFP anode provide any Al^3+^, which imply the capacities of CFP/HOTF-H_2_O/Mn_3_O_4_ and CFP/HOTF-H_2_O/Al_*x*_MnO_2_·*n*H_2_O are totally attributed to H_3_O^+^. As shown in Fig. 5b–d, both the CFP/HOTF-H_2_O/Al_*x*_MnO_2_ and CFP/HOTF-H_2_O/Mn_3_O_4_ cells show long charge plateau of 0.81 V, however, without any discharge capacity. Therefore, other cations including H_3_O^+^ in the electrolyte do not contribute to the discharge capacities at all and all the discharge capacity of the Al/Al(OTF)_3_-H_2_O/Al_*x*_MnO_2_·*n*H_2_O results from Al^3+^ intercalation.

If Al^3+^ ions are not sufficient in coin cell, how does the electrochemical performance of coin cell change? To clarify this point, the Al/HOTF-H_2_O/Mn_3_O_4_ cell was investigated. Considering no Al^3+^ resource in HOTF electrolyte, the Al anode must be adopted to help provide a small amount of Al^3+^ by dissolving Al foil with the acidic HOTF. As shown in Fig. 5c, e, owing to the dissolved Al^3+^, it shows discharge capacity of 30 mAh g^−1^ and a charge plateau of 1.4 V which is much closer to the Al/Al(OTF)_3_-H_2_O/Al_*x*_MnO_2_·*n*H_2_O cell. The results indicate that the trivalent Al^3+^ directly lead to the typical charge and discharge behavior.

To further confirm the Al^3+^ intercalation occurs during discharge, the TEM-EDS is employed to quantify Al, Mn concentration in the Mn_3_O_4_, Al_*x*_MnO_2_·*n*H_2_O and the cathode materials after discharge (Supplementary Figure 7). As shown in Table 2, the atomic ratio of Al/Mn is 0.1066 in Al_*x*_MnO_2_·*n*H_2_O after the formation process, the atomic ratio of Al/Mn increases to 0.5456 after discharge. Since the cathode materials (Al_*x*_MnO_2_·*n*H_2_O) are formed in-situ from Mn_3_O_4_ precursor and never removed from the cell during the electrochemical performance test, the specific capacity is calculated based on the weight of Mn_3_O_4_. Thus, with the molecular weight of Mn_3_O_4_, the variation of atomic ratio of Al/Mn, the theoretical capacity ascribed to Al^3+^ intercalation can be calculated according to the equation:

1 $${\mathrm{Al}}_x{\mathrm{MnO}}_{\mathrm{2}}{\mathrm{\cdot }}n{\mathrm{H}}_{\mathrm{2}}{\mathrm{O}} + {\mathrm{3}}\left( {{y} - {x}} \right){e}^ - + \left( {{y} - {x}} \right){\mathrm{Al}}^{{\mathrm{3}} + } \to {\mathrm{Al}}_y{\mathrm{MnO}}_{\mathrm{2}}{\mathrm{\cdot }}n{\mathrm{H}}_{\mathrm{2}}{\mathrm{O}}$$

**Table 2:** The Al/Mn atomic ratios of the nanoparticles obtained from the cathode charged and discharged to various states

Cathode materials charged and discharged to various states	Atomic ratio of Al/Mn
Mn_3_O_4_	0
Al_*x*_MnO_2_·*n*H_2_O	0.1066
Al_*y*_MnO_2_·*n*H_2_O (after first galvanostatic discharge)	0.5456

The expected reversible discharge capacity is 462 mAh g^−1^, which is in good agreement with our experimental data of 467 mAh g^−1^, suggesting the trivalent intercalation of Al^3+^ dominants the discharge reaction. On the anode side, weight loss which is in proportion to the discharge depth (Supplementary Table 3), and corrosive pit (Supplementary Figure 8) were observed on the Al foil, implying that the Al^3+^ intercalated in the cathode materials are derived from the striping of Al anode.

As for the charging process, there must be extraction of cations or proton generation from cathode side to balance the charge. Taking all possible cations or protons in Al/Al(OTF)_3_-H_2_O/Al_*x*_MnO_2_·*n*H_2_O into consideration, the possible reactions are as follows:

2 $${\mathrm{Al}}_y{\mathrm{MnO}}_{\mathrm{2}}\cdot n{\mathrm{H}}_{\mathrm{2}}{\mathrm{O}} - {\mathrm{3}}\left( {{y} - {x}} \right)e \to {\mathrm{Al}}_x{\mathrm{MnO}}_{\mathrm{2}}\cdot n{\mathrm{H}}_{\mathrm{2}}{\mathrm{O}} + \left( {y - x} \right){\mathrm{Al}}^{{\mathrm{3}} + }$$

3 $${\mathrm{Al}}_y{\mathrm{MnO}}_{\mathrm{2}}\cdot n{\mathrm{H}}_{\mathrm{2}}{\mathrm{O}} - {\mathrm{2}}\left( {{\mathrm{1}} - k} \right)e \to {\mathrm{Al}}_x{\mathrm{Mn}}_k{\mathrm{\cdot }}n{\mathrm{H}}_{\mathrm{2}}{\mathrm{O}} - \left( {{\mathrm{1}} - k} \right){\mathrm{Mn}}^{{\mathrm{2}} + }$$

4 $${\mathrm{Al}}_y{\mathrm{MnO}}_{\mathrm{2}}\cdot n{\mathrm{H}}_{\mathrm{2}}{\mathrm{O}} - {{2}}e \to {\mathrm{Al}}_x{\mathrm{MnO}}_{\mathrm{3}}\cdot \left( {n - {\mathrm{1}}} \right){\mathrm{H}}_{\mathrm{2}}{\mathrm{O}} + {\mathrm{2H}}^ +$$

According to the experiment phenomena, the extraction of Al^3+^ (equation (2)) is the main reaction in charging process. Even though Mn^2+^ extraction (equation (3)) and proton generation (equation (4)) are also possible during charging, they do not dominate the charge process, the reasons are discussed as follows.

If the extraction of Mn^2+^ (equation (3)) dominates the charging process, after charging for 500 mAh g^−1^ in the 2nd cycle, the cathode should lose 30% Mn in the host materials, and the capacity would decline to 0 after 5 cycles. If the proton generation (equation (4)) is indeed dominating over other reactions, the valence states of Mn and standard potential of cathode/Al would increase along with the charging process. However, in the discharge process, the proton insertion does not take place to lower the valence state of Mn. As a result, the discharge and charge plateau would be higher and higher every cycle. Considering the highest valence of Mn (VII), the highest charging capacity that the cathode can afford is 1300 mAh g^−1^, which means the charging process would never survive than 3 cycles. Obviously, neither of the above phenomena occurs in the cycles. Hence, it is reasonable to believe that the extraction of Al^3+^ (equation (2)) contributes most to the charging capacity. Indeed, equations (3) and (4) may occur as side reaction. Since both of them are cathode consuming processes, they may be the cause of capacity decay. Thus, suppression of these side reactions is a possible strategy to further enhance the stability of this aqueous aluminum-ion battery.

All the above results infer that the insertion/extraction of trivalent Al^3+^ dominates the electrochemical reaction. Besides, the typical electrochemical performance of high capacity and plateaus is mainly attained by the reversible intercalation of trivalent Al^3+^. Furthermore, to probe the benefits of solvent water in aqueous electrolyte Al(OTF)_3_-H_2_O and the crystal water in cathode materials Al_*x*_MnO_2_·*n*H_2_O, the ionic liquid AlCl_3_/[BMIM]Cl (mole ratio 1.1:1) (H_2_O-free) is employed as a comparison electrolyte. It has been proven that the Al^3+^ can be electrochemically deposited from AlCl_3_/[BMIM]Cl and intercalate into the cathode materials^8^. But this ionic liquid is ultra-dry with only ions ([BMIM]^+^ and Al_*x*_Cl_*y*_^−^^44^) and absent from aqueous solvation (Table 1). Because the spinel-to-layered reaction only occurs in aqueous solution^32,33,35,37,42^, the hydrous mixed phase Al_*x*_MnO_2_·*n*H_2_O should not be formed in ionic liquid.

The spinel Mn_3_O_4_ displays a capacity of 130 mAh g^−1^ in its first discharge with a plateau lower than 0.5 V. However, the capacity decreases to 18 mAh g^−1^ after 20 cycles. Using the same electrolyte (the ionic liquid AlCl_3_/[BMIM]Cl), the Al_*x*_MnO_2_·*n*H_2_O not only delivers a much higher reversible capacity of 229 mAh g^−1^ during first discharge, but also maintains the capacity of 55 mAh g^−1^ after 50 cycles (Fig. 5b, d). The much higher discharge capacity and retention capabilities of Al_*x*_MnO_2_·*n*H_2_O over spinel Mn_3_O_4_ is reasonable due to the superior layered structure for ion intercalation and the shielding effects from crystal water to the electrostatic interaction between the Al^3+^ and the host anions.

Nevertheless, the electrochemical performance of the Al_*x*_MnO_2_·*n*H_2_O in the ionic liquid AlCl_3_/[BMIM]Cl electrolyte is still much poorer than the Al(OTF)_3_-H_2_O electrolyte (Fig. 5b, d). This phenomenon outstands the merits of the solvent water. Because of its high valent states and small diameter, the Al^3+^ is easily trapped in lattice and defects of host materials and extraction of Al^3+^ from the cathode materials always lead to striking overpotential, poor cycling ability^8^. This sluggish kinetic is mitigated in this aqueous electrolyte. The charging profiles of the Al/Al(OTF)_3_-H_2_O/Al_*x*_MnO_2_·*n*H_2_O (Fig. 5a) and electrochemical transformation (Supplementary Figure 2c) appear to be similar. This suggests that the charging process is similar to the electrochemical transformation process, which is a complicated structural rearrangement of the materials involving H_2_O insertion^34–36^. During this process, the Al^3+^ is extracted from the cathode material and solvated by water. The solvent H_2_O insert into the host framework and form the crystal water in the cathode materials^35^, which help to shield the strong electrostatic force between Al^3+^ and host frame. As a result, the Al^3+^ is electrochemically extracted from the Al_*y*_MnO_2_·*n*H_2_O with much faster kinetics and higher reversible capacity. The AlCl_3_/[BMIM]Cl, on the other hand, provides no water solvent molecular to form crystal water in host frame and displays the sluggish trivalent kinetics. So the cells with AlCl_3_/[BMIM]Cl electrolyte show higher overpotential and lower discharge capacity than that of cells with aqueous electrolyte (Fig. 5b). In addition, the AlCl_3_/[BMIM]Cl is so hygroscopic^31^, that it will tend to consume the crystal water in the Al_*x*_MnO_2_·*n*H_2_O. The layered structure with crystal water will likely collapse in this ionic liquid, which results in serious decay in the capacity. All the phenomena indicate that the AlCl_3_/[BMIM]Cl is less compatible with the cathode materials with strong bond in host frame. Hence, the different electrochemical performance of Al/Al(OTF)_3_-H_2_O/Al_*x*_MnO_2_·*n*H_2_O and Al/AlCl_3_,BMIMCl/Al_*x*_MnO_2_·*n*H_2_O indicate that the aqueous electrolyte is critical in mitigating the sluggish kinetics of trivalent reaction and maintaining the crystal water in the structure of the cathode.

## Discussion

An aqueous rechargeable aluminum-ion battery is assembled with a promising key cathode material Al_*x*_MnO_2_·*n*H_2_O, prepared through in-situ method of electrochemical transformation from spinel to layered and amorphous mixed phase for the first time. The Al_*x*_MnO_2_·*n*H_2_O delivers a specific capacity of 467 mAh g^−1^ and energy density of 481 Wh kg^−1^, both of which are among the highest values achieved in rechargeable aluminum-ion batteries. A series of comparative experiments reveal the respective roles of the Al^3+^ ion and aqueous solution in achieving high electrochemical performance. The dominant role of Al^3+^ insertion/extraction in electrochemical reaction is confirmed by comparing the electrochemical behavior of cell using Al(OTF)_3_-H_2_O and HOTF-H_2_O as the electrolyte. The aqueous electrolyte is found to be crucial for improving kinetics and maintaining the cyclic durability by comparing the electrochemical performance of Al(OTF)_3_-H_2_O and AlCl_3_/[BMIM]Cl ionic liquid. The satisfactory performance of this Al/Al(OTF)_3_-H_2_O/Al_*x*_MnO_2_·*n*H_2_O could be ascribed to Al_*x*_MnO_2_·*n*H_2_O accommodating the Al^3+^, producing both the high voltage and the superior capacity, as well as faster kinetics by using aqueous electrolyte.

The outstanding energy density, low cost, facile cell assembly along with the important safety implications of an aqueous electrolyte, make this aqueous aluminum-ion battery promising for large-scale energy storage application. Moreover, the strategy of electrochemical transformation and the design of tailoring polarity should lead the way to explore more transition metal oxides as electrode materials and develop cathode materials with higher energy density for rechargeable aluminum-ion batteries.

## Methods

### Synthesis of the materials and preparation of the Al/Al(OTF)_3_-H_2_O/Al_*x*_MnO_2_·*n*H_2_O coin cell

Mn_3_O_4_ nanoparticles were synthesized by deposition method, the 4 mmol manganese acetate was dissolved into 60 mL deionized water, 9.5 mmol hydrazine hydrate was added into the solution dropwise with stirring in 25 °C. The Mn_3_O_4_ nanoparticles were dried in vacuum at 60 °C for 12 h and calcined in Argon at 200 °C for 5 h. The as-prepared Mn_3_O_4_ nanoparticles was ground together with Super-P and polytetrafluoroethylene (PTFE) binder (mass ratio, Mn_3_O_4_: super-P: binder = 8:1:1). The slurry was spread on carbon fiber paper (Toray, H90) for electrochemical performance or Mo foil for ex-situ XRD characterization and dried at 60 °C for 12 h.

Two types of transparent aqueous electrolytes with the concentration (5 and 3.16 mol L^−1^) were obtained by dissolving Al(OTF)_3_ (99%, Acros Chemicals) and trifluoromethanesulfonic acid (HOTF, 99%, Acros Chemicals) into dilute water, respectively. The aqueous electrolyte is identified as Al(OTF)_3_-H_2_O and HOTF-H_2_O. The ionic liquid electrolyte was prepared by dissolving aluminum chloride (AlCl_3_, 99.99% Acros Chemicals) into 1-butyl-3-methylimidazolium chloride ([BMIM]Cl, 98% Acros Chemicals) with a mole ratio of 1.1:1.

Al_*x*_MnO_2_·*n*H_2_O was prepared by galvanostatic electrochemical transformation in aqueous electrolyte (5 mol L^−1^ Al(OTF)_3_-H_2_O electrolyte). For this, the Mn_3_O_4_ electrodes were assembled into the 2025 coin cells with the Al(OTF)_3_-H_2_O (5 mol L^−1^) electrolyte, a Whatman glass fiber (GF/C) as the separator, and Al foil counter electrode. Then the cells were galvanostatic charged to 1.8 V (vs. Al/Al^3+^) in the Al(OTF)_3_-H_2_O under the current density of 30 mA g^−1^. During this charging process, spinel Mn_3_O_4_ was progressively transformed into Al_*x*_MnO_2_·*n*H_2_O.

### Materials characterization

The Mn_3_O_4_ nanoparticles and cathodes for ex-situ tests were characterized by X-ray powder diffraction (XRD, Rigaku Ultima IVD/MAX-RB) with Cu Kα radiation (*λ* = 0.15406 nm) at ambient temperature. For the ex-situ XRD tests, the cathodes were removed from various charge states in the glove box, washed with deionized water and ethanol for three times respectively, and dried in vacuum. The field-emission scanning electron microscopy (HITAS-4800), HRTEM, and energy-dispersive microscopy (Hitachi H-800) were employed to investigate the morphologies, microstructure, and element contention variation of the cathode materials. The HAADF and ABF imaging of STEM was performed using a spherical aberration-corrected electron microscope at an acceleration voltage of 200 kV (JEM-ARM 200F transmission electron microscope). The HAADF and ABF images were taken simultaneously with two detectors of HAADF and ABF imaging which are aligned along the optical axis.

### Electrochemical measurements

2025 coin-type cells were assembled for the electrochemical performance tests using Whatman glass fiber as separator. The cells of various combinations of cathode materials, electrolyte, and anode were simplified as anode/electrolyte/cathode (e.g., Al/Al(OTF)_3_-H_2_O/Al_*x*_MnO_2_·*n*H_2_O). The cells using electrolyte of Al(OTF)_3_-H_2_O and HOTF-H_2_O were assembled in air, the cells using electrolyte of ionic liquid AlCl_3_/[BMIM]Cl were assembled in an Ar-filled glove box.

Galvanostatic discharge/charge measurements were conducted on a LAND battery system (CT2001A, Wuhan, China) in the voltage range of 0.5–1.8 V (vs. Al) at 30 °C The CV tests were performed on CHI 606D electrochemical workstation. In the electrochemical windows investigations, the glassy carbon electrode (diameter: 2 mm) was used as the working electrode, and Al metal foil was used as the counter electrode, the Ag/AgCl electrode was used as reference electrodes. The CV scan rate is 10 mV s^−1^. The symmetric cell was assembled by using Al as cathode and anode, the aqueous Al(OTF)_3_ (5 mol L^−1^) as the electrolyte. The current density of galvanostatic cycling for symmetric cell is 0.01 mA cm^−2^.

### Computational methods

Our geometry optimizations were performed within the Cambridge Serial Total Energy Package (CASTEP), based on density functional theory (DFT). The exchange-correlation functional energy was processed by the Perdew–Burke–Ernzerhof (PBE) function within the generalized gradient approximation (GGA). The cut-off energy used for the plane wave expansion of the wave function was 500 eV. A dense Monkhorst–Pack k-points 3 × 1 × 3 was used for the Brillouin zone. All the compounds were fully relaxed until the differences of the total energy were less than 1.0 × 10^−5^ eV, and atomic force were within 1 × 10^−3^ eV.

### Calculations

The theoretical capacity of Al/Al(OTF)_3_-H_2_O/Al_*x*_MnO_2_·*n*H_2_O can be calculated according to the equation of *C* = *nF*/3.6*M*. Because the Al_*x*_MnO_2_·*n*H_2_O is formed in situ on the cell and never be removed, so the capacity was calculated based on the weight of the precursor Mn_3_O_4_. In the first charge process, the Mn_3_O_4_ transformed into Al_*x*_MnO_2_·*n*H_2_O, and the discharge reaction was as follows:

$${\mathrm{Al}}_x{\mathrm{MnO}}_{\mathrm{2}}\cdot n{\mathrm{H}}_{\mathrm{2}}{\mathrm{O}} + {\mathrm{3}}\left( {y - x} \right)e^ - + \left( {y - x} \right){\mathrm{Al}}^{{\mathrm{3 + }}} \to {\mathrm{Al}}_y{\mathrm{MnO}}_{\mathrm{2}}\cdot n{\mathrm{H}}_{\mathrm{2}}{\mathrm{O}}$$

The *n* is calculated based on the variation of Al/Mn contents in Table 2, and the reduction of per Mn is accompanied by 3(*y* − *x*)*e*^−^ = 3(0.5456 − 0.1066)*e*^−^ = 1.317*e*^−^. Since the capacity was calculated based on Mn_3_O_4_, the charge transfer calculated per Mn_3_O_4_ is 3 × 1.317*e*^−^ = 3.951*e*^−^, then the theoretical capacity was calculated as follows:

$${C} = \frac{{\left( {0.5456 - 0.1066} \right) \times 3 \times 3 \times 96,485\left( {{\mathrm{c}}\,{\mathrm{mol}}^{ - 1}} \right)}}{{3.6 \times 228.8\left( {{\mathrm{g}}\,{\mathrm{mol}}^{ - 1}} \right)}} = 462\,{\mathrm{mAh}}\,{\mathrm{g}}^{ - 1}$$

Theoretically, the anode weight loss is calculated according to the Faraday’s First Law of Electrolysis:

$$\begin{array}{c}m = MQ{\mathrm{/}}nF\\ = \left[ {m_{\left( {{\mathrm{cathode}}\,{\mathrm{materials}}} \right)} \times n_{\left( {{\mathrm{cathode}}\,{\mathrm{materials}}} \right)} \times M_{\left( {{\mathrm{Al}}} \right)}} \right]{\mathrm{/}}\left[ {M_{\left( {{\mathrm{cathode}}\,{\mathrm{materials}}} \right)} \times n_{\left( {{\mathrm{Al}}} \right)}} \right].\end{array}$$

## Supplementary information

Supplementary Information

## Data Availability

The data that support the findings of this study are available from the corresponding authors upon request.
