# Supplementary material for: Electrochemically activated spinel manganese oxide for rechargeable aqueous aluminum battery
Source: Nat Commun. 2019 Jan 8;10:73. doi: 10.1038/s41467-018-07980-7 (PMC6325165; doi:10.1038/s41467-018-07980-7)
Supplement: Supplementary file 1 — Supplementary Information [file 41467_2018_7980_MOESM1_ESM.docx]

**Supporting Information**

Electrochemically Activated Spinel Manganese Oxide for Rechargeable Aqueous Aluminum Battery

Wu et al.

## Supplementary Figures


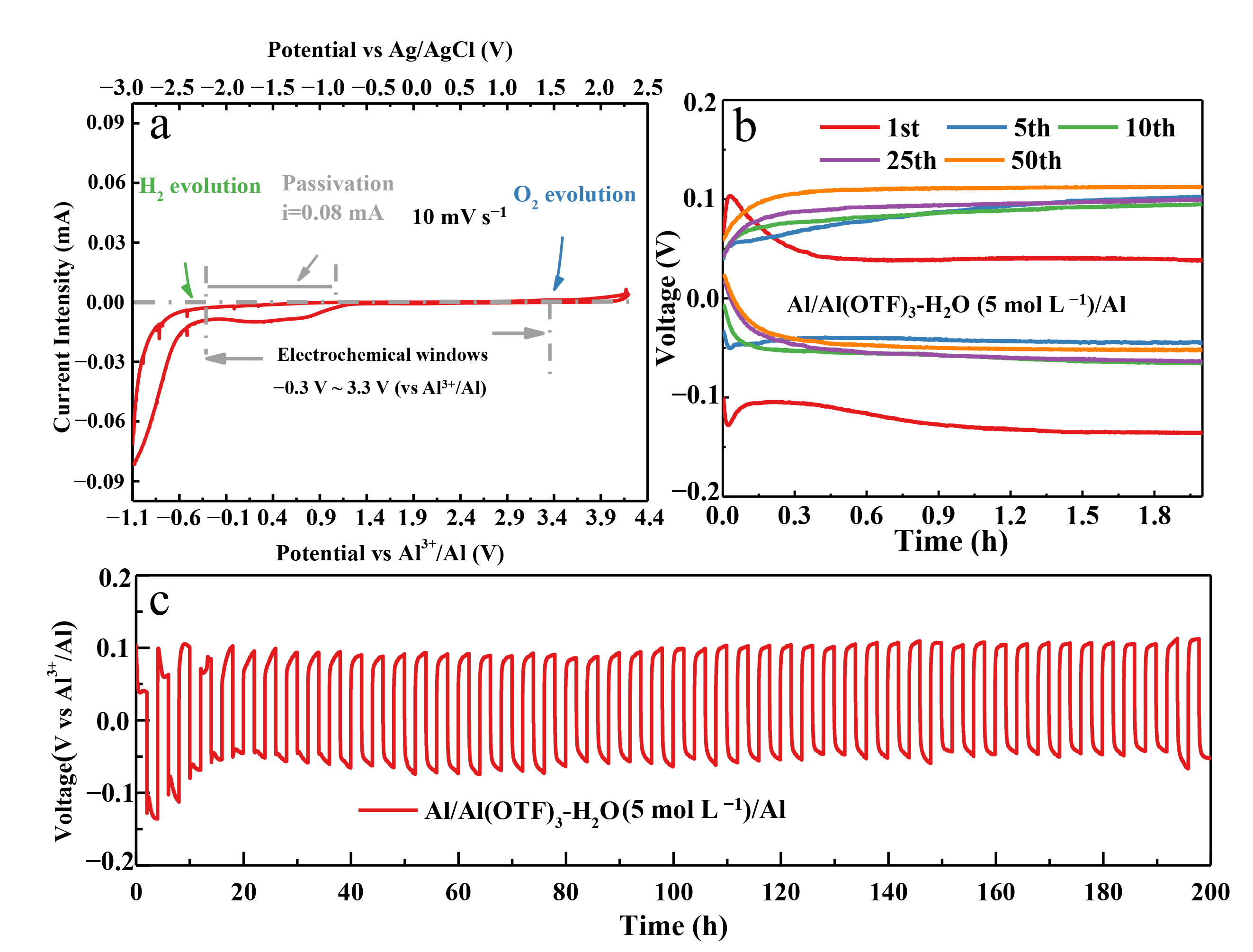


**Supplementary Figure 1**. **Stable electrochemical window of Al(OTF)_3_ (5 mol L^−1^) aqueous solution. a** CV test for electrochemical active window of aqueous Al(OTF)_3_ (5 mol L^−1^) solution using Glass carbon as working electrode, Ag/AgCl as the reference, the Al as the counter electrode, the potential is converted to Al^3+^/Al for convenience; **b** Galvanostatic charge and discharge profile for Al-Al symmetric cell with aqueous Al(OTF)_3_ (5 mol L^−1^) electrolyte; **c** Profile of voltage versus time


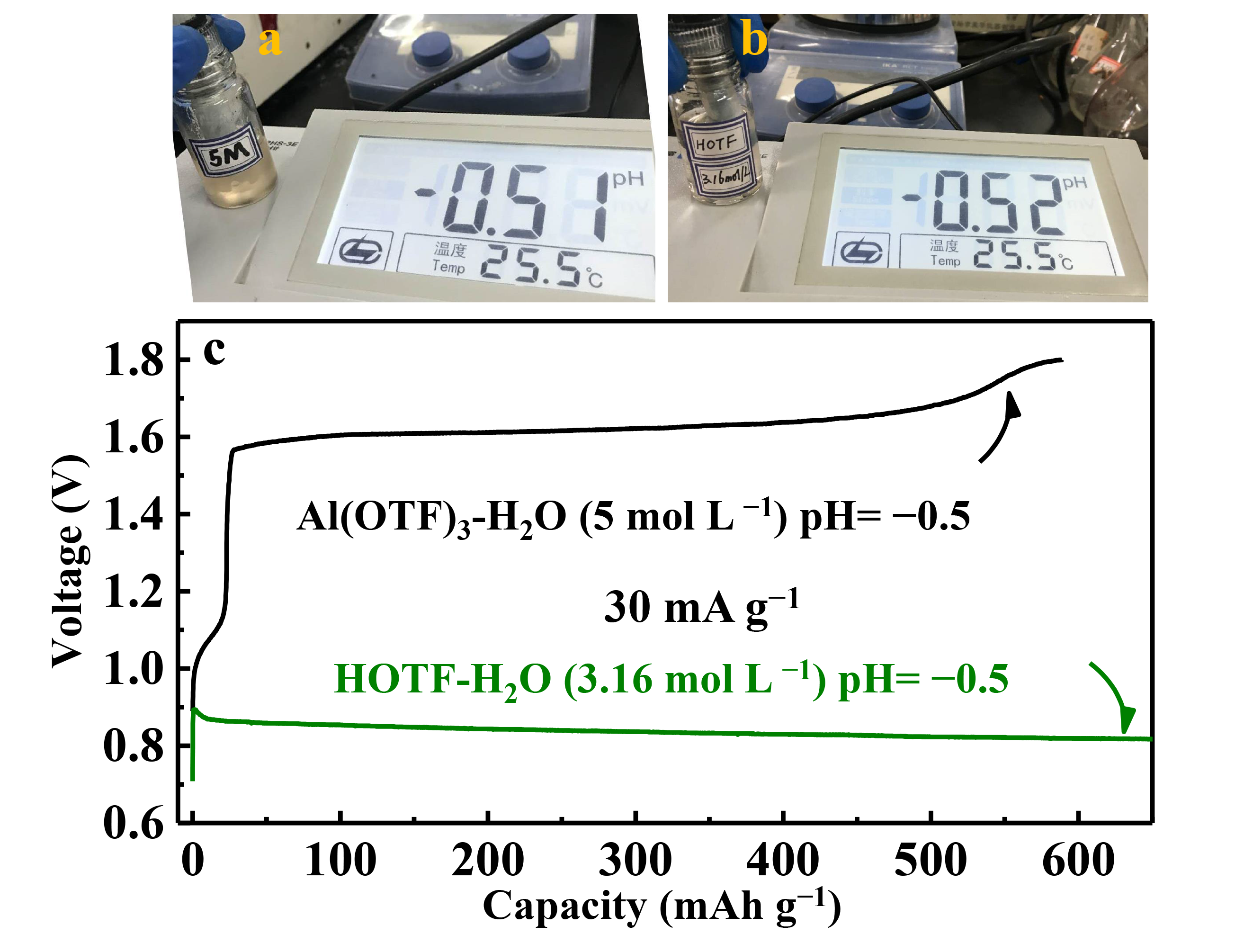


**Supplementary Figure 2**. **Al^3+^ effects on the galvanostatic charge profile.** **a** aqueous Al(OTF)_3_ (5 mol L^−1^) solution and **b** aqueous HOTF (3.16 mol L^−1^); **c** Galvanostatic charge profiles of the electrochemical transformation process in aqueous Al(OTF)_3_ (5 mol L^−1^) electrolyte and aqueous HOTF (3.16 mol L^−1^) electrolyte


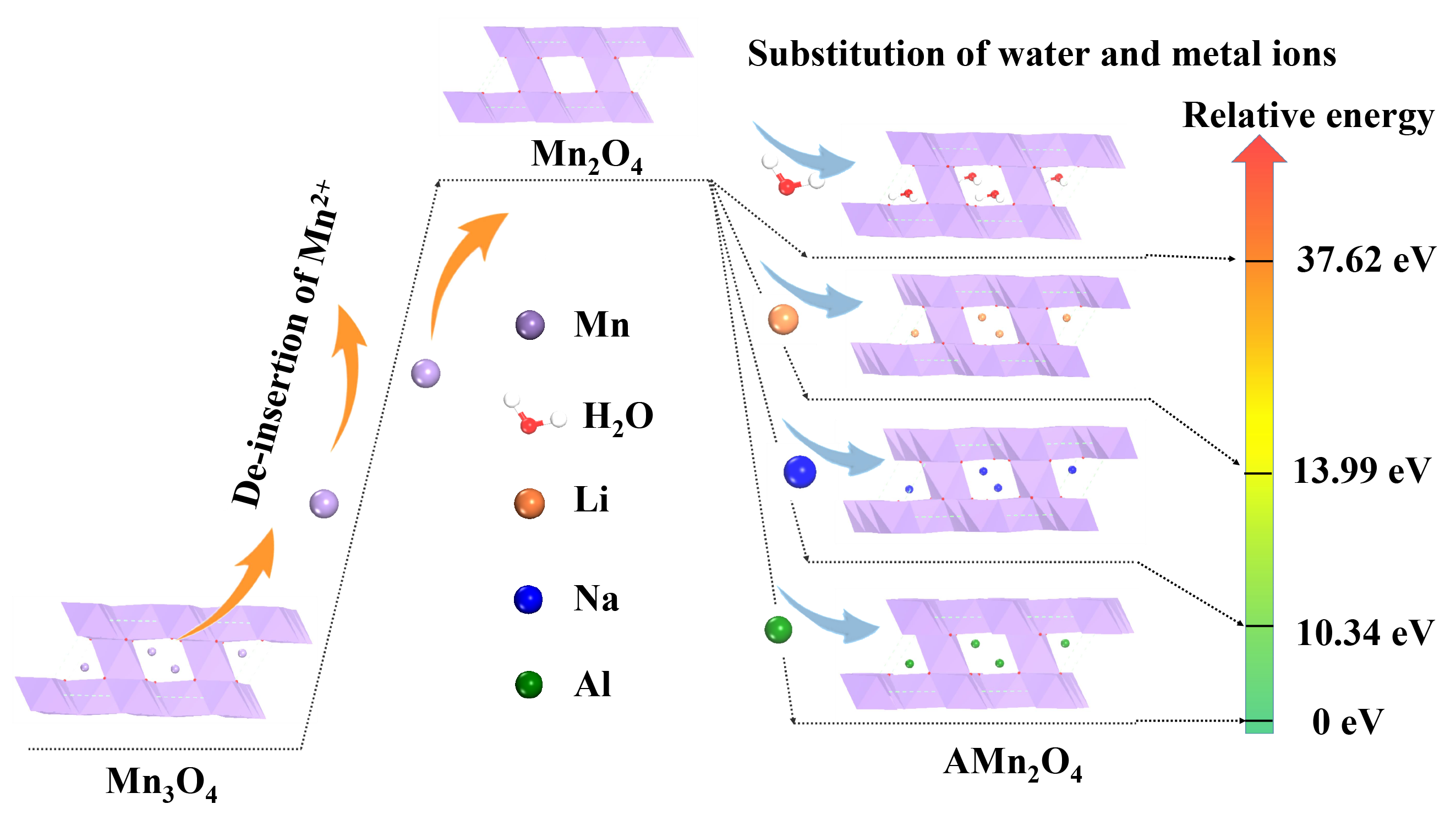


### Supplementary Figure 3. Scheme of crucial step in spinel-to-layered phase transition and the stability of H_2_O or charge carriers intercalated metastable A_x_Mn_2_O_4_. The relative energy were defined as ΔE =E(A_x_Mn_2_O_4_)−E(Mn_2_O_4_) −E(A), where x=H_2_O, Al^3+^, Li^+^ or Na^+^, the relative energy were displayed at right side


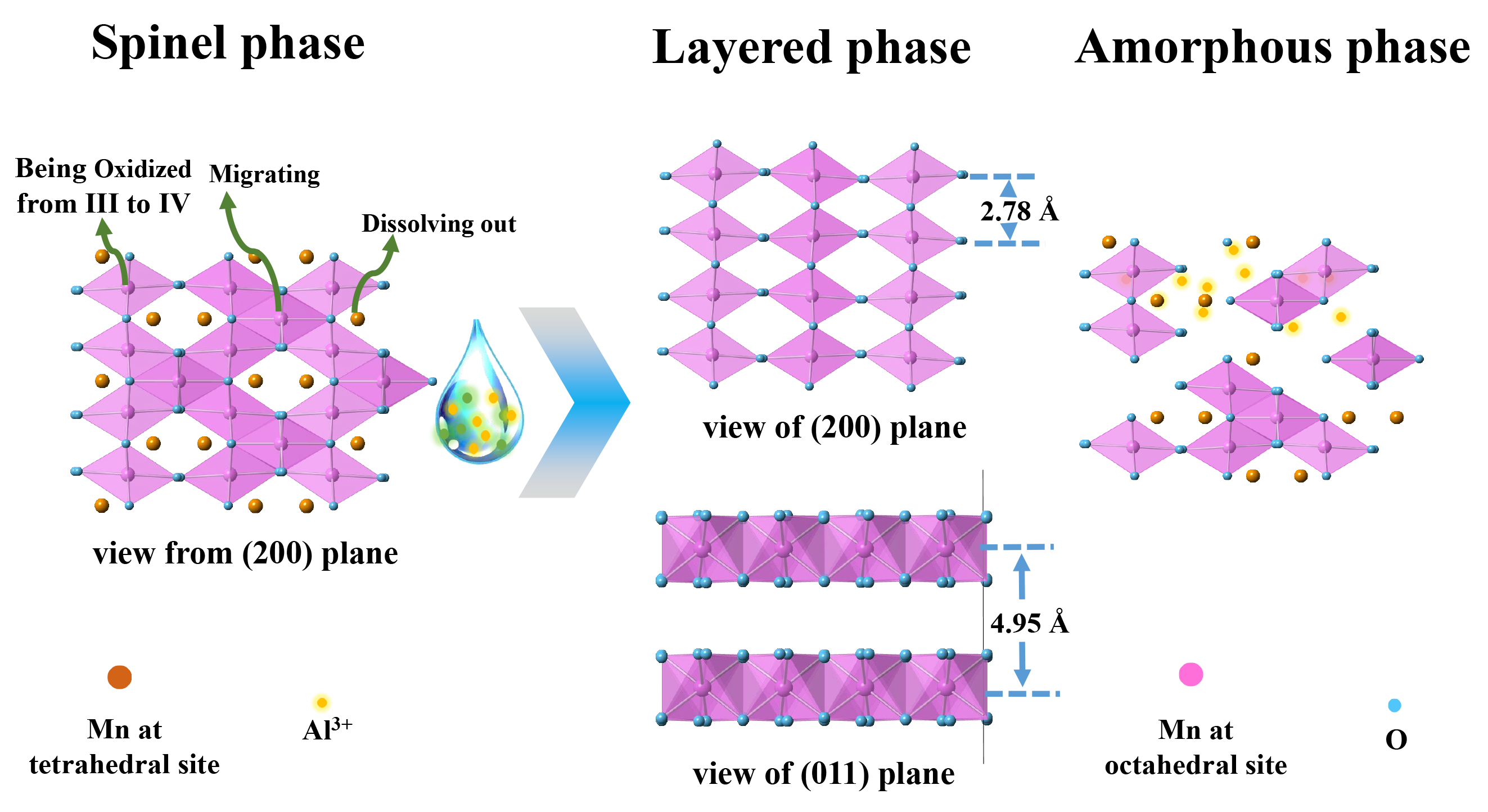


**Supplementary Figure 4.** **Scheme for the structure evolution from spinel Mn_3_O_4_ to layered phase and amorphous phase**


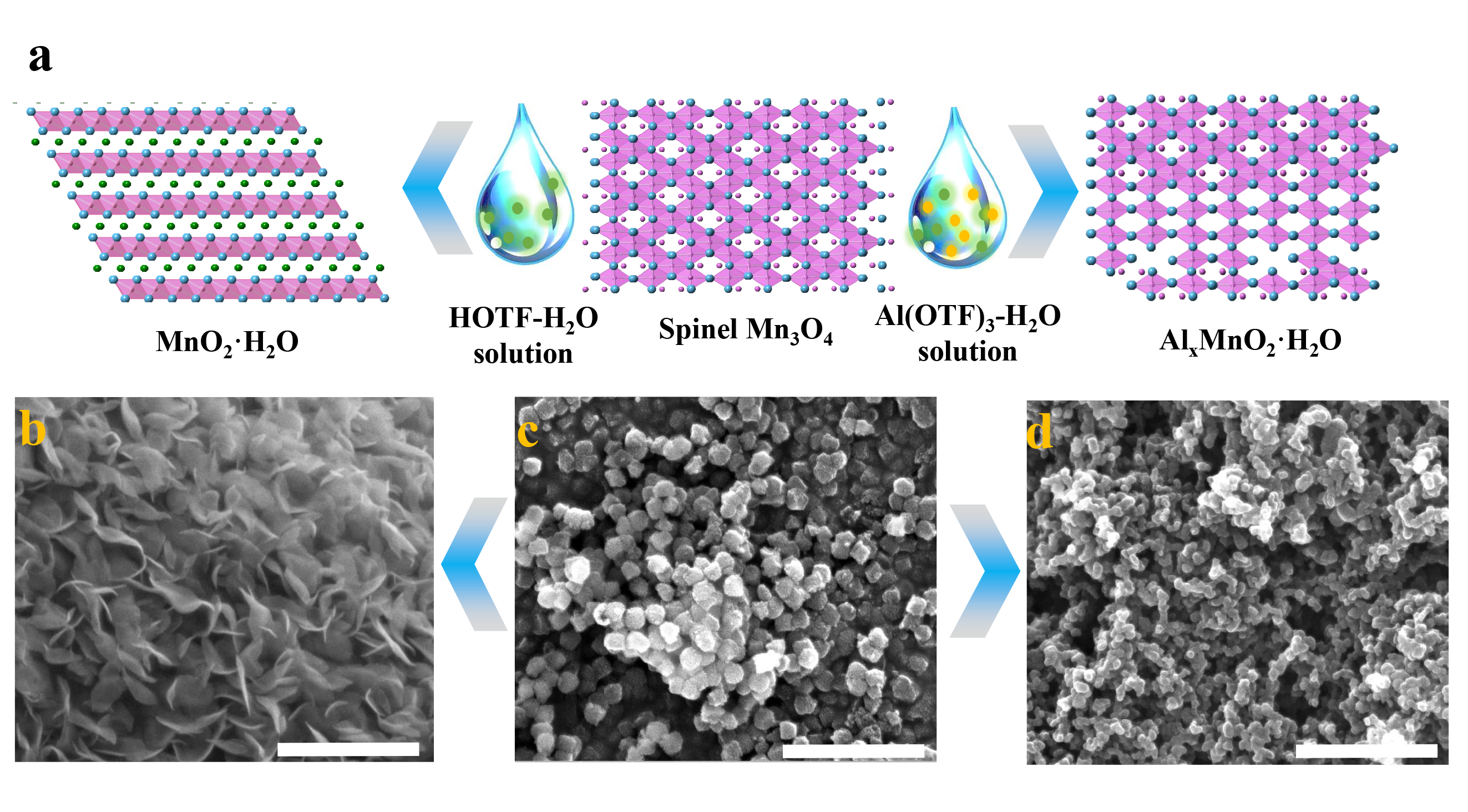


**Supplementary Figure 5. The Al^3+^ effects on the morphology of products. a** Illustration structures of the electrochemical transformation resultant samples in different electrolyte; **b** SEM of the sample transformed in aqueous HOTF (3.16 mol L^−1^) solution; **c** SEM of pristine spinel Mn_3_O_4_; **d** SEM of the sample transformed in Al(OTF)_3_-H_2_O (5 mol L^−1^) solution. Scale bar: 1 um for **b**, **c** and **d**


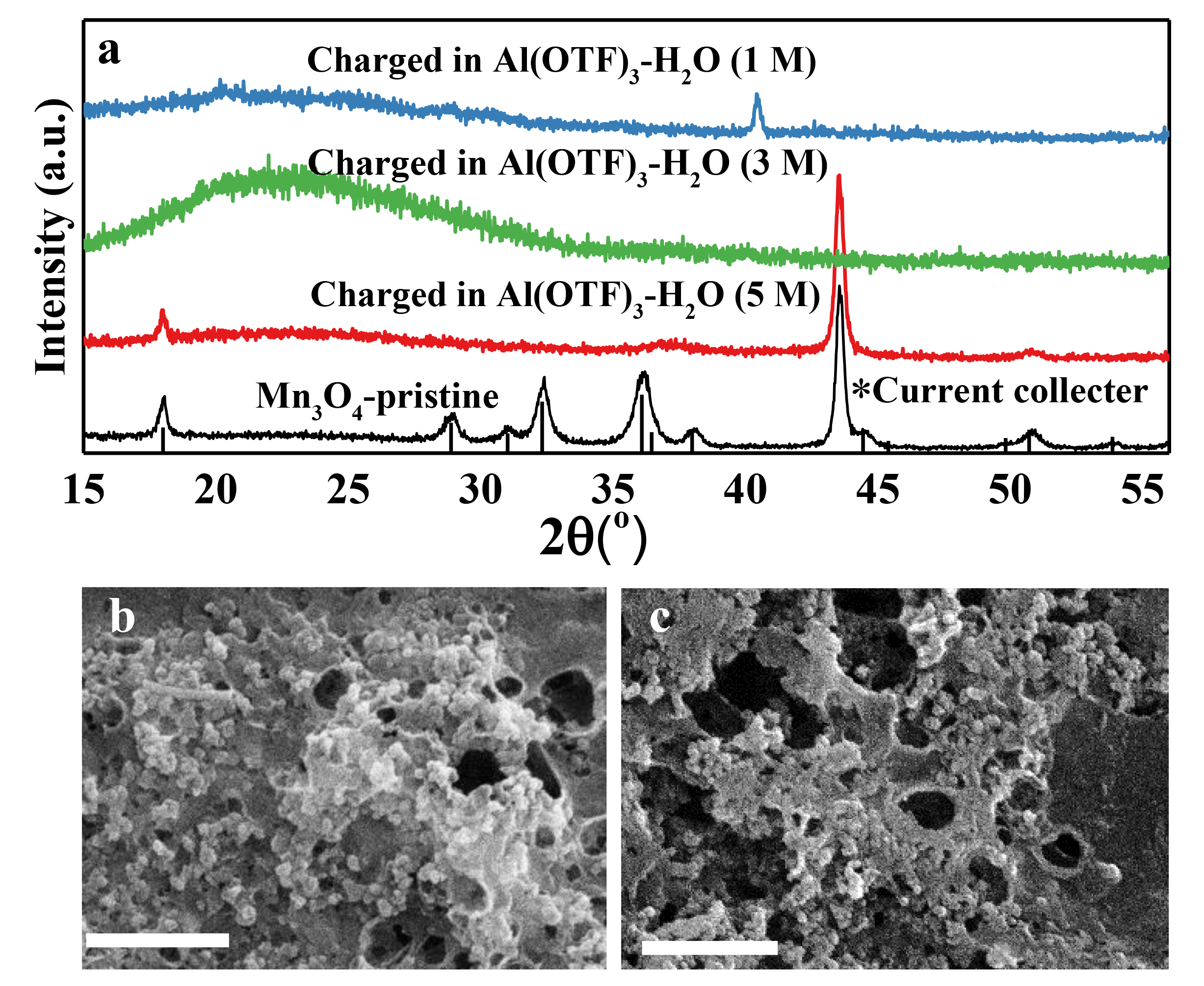


### Supplementary Figure 6. The concentraion effects of Al^3+^ on the formation of Al_x_MnO_2_·*n*H_2_O. a XRD patterns of the samples formed in Al(OTF)_3_-H_2_O (1 M, 3 M and 5 M). b SEM of the sample formed in Al(OTF)_3_-H_2_O (1 M); c SEM of the sample formed in Al(OTF)_3_-H_2_O (3 M). Scale bar: 1 um for b and c


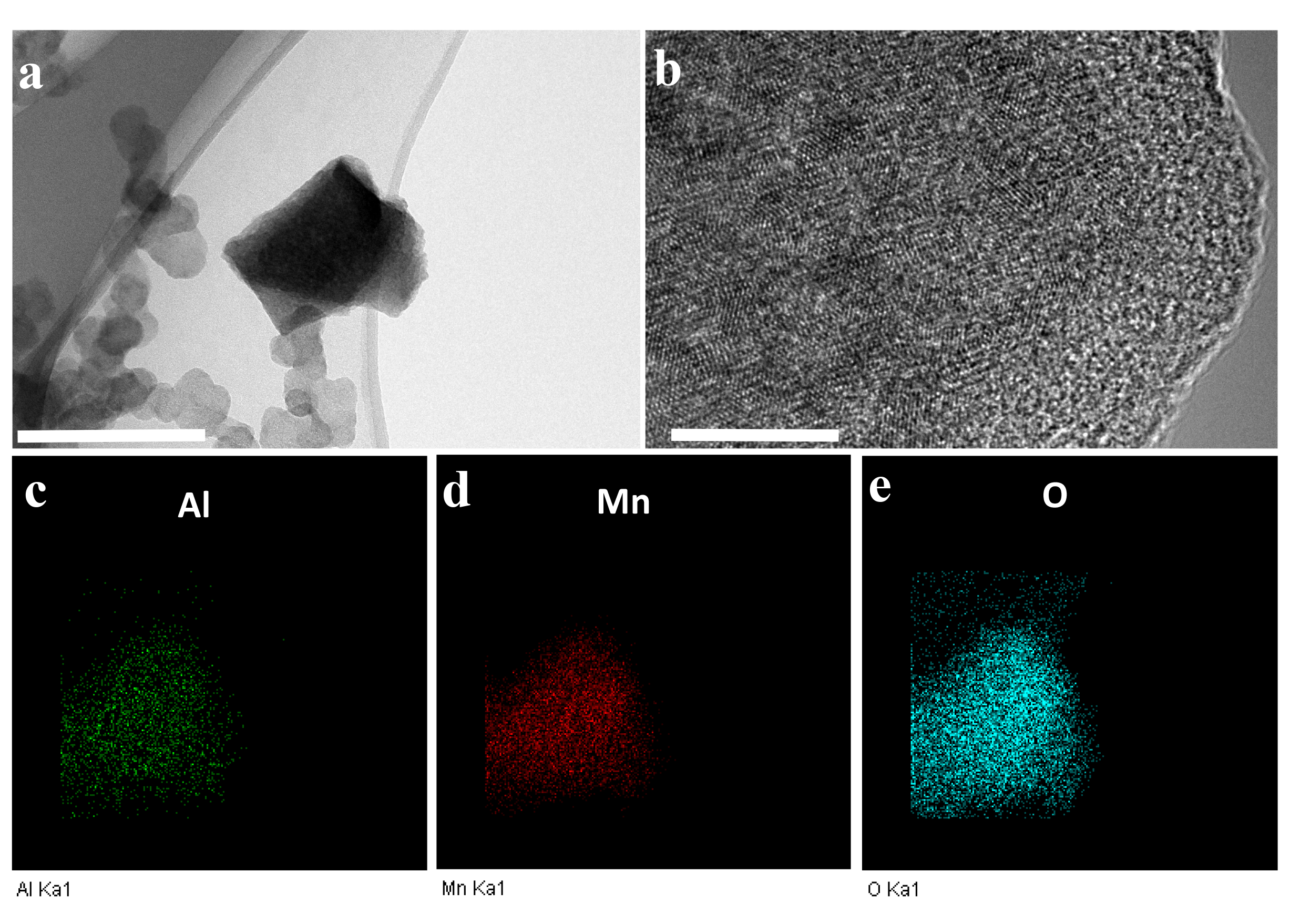


**Supplementary Figure 7. The TEM of cathode mateials after discharge. a,** **b** TEM image of Al_x_MnO_2_·*n*H_2_O nanoparticles after discharge; **c-e** element mapping of Al_x_MnO_2_·*n*H_2_O after discharge: c Al, d Mn, e O. Scale bar: 200 nm for **a** and 10 nm for **b**


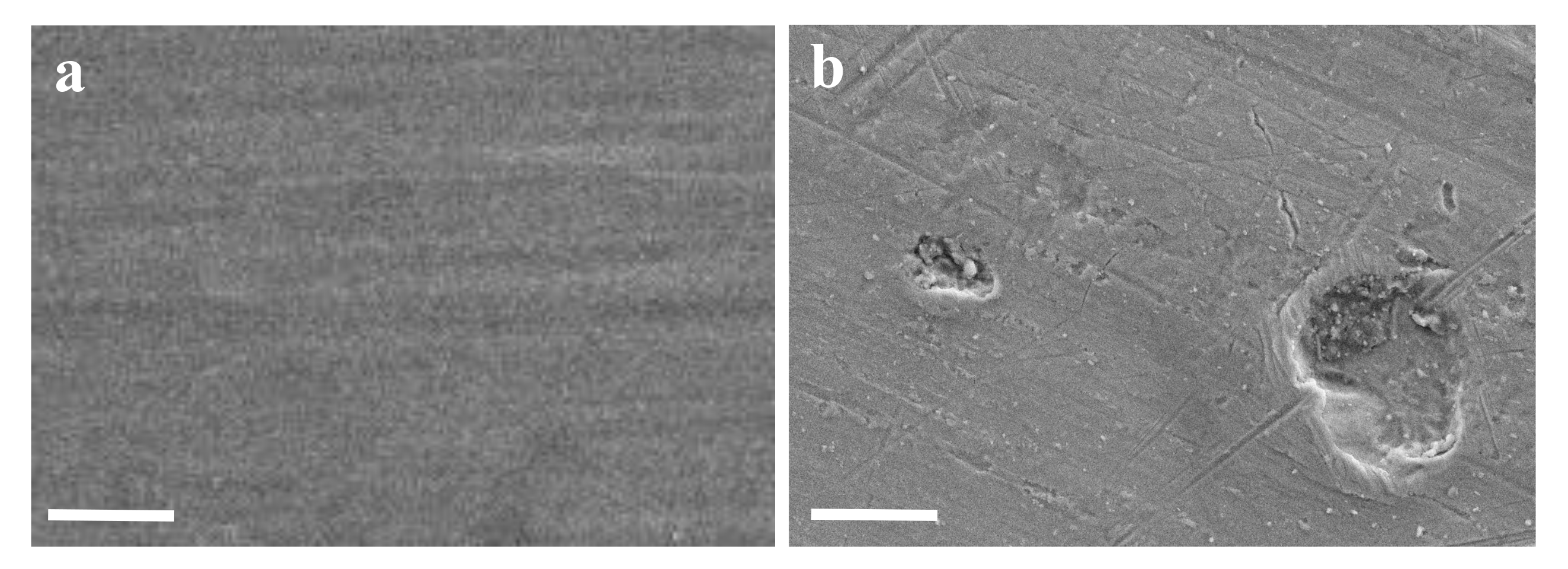


**Supplementary Figure 8. The SEM of Al foil before and after discharge.** **a** Al foil before discharge, **b** Al foil after discharge. Scale bar: 10 um for **a** and **b**

## Supplementary Tables

**Supplementary Table 1**. The ions species in the electrochemical transformation coin cells with Al(OTF)_3_-H_2_O (5 mol L^−1^) and HOTF-H_2_O (3.16 mol L^−1^) electrolyte

| electrolyte | cathode materials  (precursor) | anode | ions species |
| --- | --- | --- | --- |
| Al(OTF)_3_-H_2_O (5 mol L^−1^) | Mn_3_O_4_ | Al | Al^3+^, H_3_O^+^, OTF^−^ |
| HOTF-H_2_O (3.16 mol L^−1^) |  | CFP | H_3_O^+^, OTF^−^ |

**Supplementary Table 2.** Electrochemical performance of reported rechargeable aluminum ion batteries based on trivalent reaction mechanism

| Cathode materials | Discharge capacity  (mAh g^−1^)^a^  /current density (mA g^−1^) | Discharge potential (V) | Capacity retention (after 20 and 50 cycles)^c^ | Reference |
| --- | --- | --- | --- | --- |
| Al_x_MnO_2_·*n*H_2_O | 467/30 | 1.1 | 72%, 58% | in this work |
| CuFe-PBA | 50/50 | 0.5 | 88%, 88% | ^1^ |
| TiO_2_ | 75/4^b^ | 0.8 | / | ^2^ |
| S | 1250/35 | 1.05 | / | ^3^ |
| FeS_2_ | 600/8.94 | 0.4 | / | ^4^ |
| CuS@C microsphere | 240/20 | 0.5 | 42%, 38% | ^5^ |
| RGO-SnS_2_ | 392/100 | 0.4 | 41%, 23 % | ^6^ |
| Ni_2_S_3_@graphene | 295/100 | 0.6 | 31% 27% | ^7^ |
| V_2_O_5_ | 303/100 | 0.55 | 90%, / | ^8^ |

^a)^ The highest discharge capacity are recorded from the corresponding reference; ^b)^ the unit of the current density is mA cm^−2^; ^c)^ If the discharge potential and capacities retention values are not indicated directly, they are estimated according to the capacity figures.

**Supplementary Table 3.** Weight loss of Al foil in varied discharge depth

| Discharge capacity(mAh g^−1^) | Experimental weight loss of Al foil (mg) | Theoretical weight loss of Al foil (mg) |
| --- | --- | --- |
| 91.3 | 0.06 | 0.05 |
| 136.8 | 0.14 | 0.07 |
| 351 | 0.32 | 0.19 |

## Supplementary Notes

### Supplementary Note 1: Determination of voltage range of electrochemical reaction.

To determine the voltage range of electrochemical reaction, the electrochemical window of Al(OTF)_3_ (5 mol L^−1^) aqueous solution was evaluated by the method of cyclic voltammetry(CV) with working electrode of glass carbon electrode, reference electrode of Ag/AgCl, and counter electrode of Al. As shown in Supplementary Figure 1a, onset of reduction occurs at 1.1 V and then reaching a plateau. This plateau indicates a passivation process^9^ that eventually suppresses hydrogen evolution in the aqueous electrolyte, pushing its reduction onset from 1.1 V to −0.3 V(vs Al^3+^/Al). Because of this passivation process, the Al can be deposited/dissolution in aqueous Al(OTF)_3_ solution without triggering hydrogen evolution.

To further confirm the feasibility of Al deposition/dissolution in this electrolyte, the symmetric Al cell with aqueous Al(OTF)_3_ was assembled. As shown in Supplementary Figure 1b, c, the symmetric cell exhibits reversible striping and plating behavior, proving that the Al foil can be used as anode directly in this aqueous Al(OTF)_3_ (5 mol L^−1^) electrolyte. On the other hand, the evolution of oxygen occurs at 3.3 V. Thus, the overall stable window for this aqueous electrolyte is determined as the voltage range of −0.3~3.3 V. Accordingly, the electrochemical transformation process was conducted in the voltage range of 0.5~1.8 V, which is within the reduction and oxidation limits of electrolyte. Thus the Al is dissolved/deposited electrochemically to balance the current without triggering any side reaction when the insertion/extraction occurs on Al anode.

### Supplementary Note 2: Al^3+^ effects on the electrochemical transformation process.

To further understand the effects of Al^3+^ in Al(OTF)_3_-H_2_O (5 mol L^−1^) on the electrochemical transformation process, we compare the Al_x_MnO_2_·*n*H_2_O with the samples formed in HOTF-H_2_O electrolyte. As shown in Supplementary Figure 2a, the pH of Al(OTF)_3_-H_2_O is −0.5, suggesting the H_3_O^+^ concentration in this aqueous electrolyte is 3.16 mol L^−1^. Owning to the weak Lewis acidity of the Al^3+^, the H_3_O^+^ is inevitably generated in the Al(OTF)_3_-H_2_O and also likely to affect the transformation reaction. To distinguish the effects of H_3_O^+^ and Al^3+^, the aqueous trifluoromethanesulfonic acid (HOTF) solution (HOTF-H_2_O) containing the same ions species of H_3_O^+^ and OTF^-^ was used as a counterpart electrolyte without Al^3+^. The H_3_O^+^ concentration of HOTF-H_2_O is 3.16 mol L^−1^, and the same pH as that of Al(OTF)_3_-H_2_O (Supplementary Figure 2 b). In assembled cell without the Al^3+^, the carbon fiber paper (CFP) was used as anode to avoid the Al^3+^ dissolving into electrolyte. Table 1 shows the ions species in the cell with Al(OTF)_3_-H_2_O and HOTF-H_2_O solution, both the Al(OTF)_3_-H_2_O and HOTF-H_2_O contain the H_3_O^+^ and OTF^−^.

Due to the only difference in containing Al^3+^ or not, it is verified that the Al^3+^ leads to the difference in electrochemical transformation. As shown in Supplementary Figure 5c, the transformation charging curve with HOTF-H_2_O electrolyte shows a charging plateau of 0.81 V, which is evidently different from the typical electrochemical transformation profile with Al(OTF)_3_-H_2_O electrolyte.

The influence of Al^3+^ on the morphology of products is shown in Supplementary Figure 5. The cubic nanoparticles evolve into lamellar structures consisting of 2D nanosheet with the thickness of about 10 nm (Supplementary Figure 5b). In comparison, the Al_x_MnO_2_·*n*H_2_O is still nanoparticles rather than nanosheet (Figure 2c, Supplementary Figure 5d). Considering the same concentration of H_3_O^+^ of those two electrolytes (Supplementary Figure 2a, Supplementary Table 1), it can be speculated that the formation of this unique mixed phase structure of Al_x_MnO_2_·*n*H_2_O is direct related to trivalent charge carriers Al^3+^.

To evaluate the effects of Al^3+^ concentration on the formation of Al_x_MnO_2_·*n*H_2_O, Al(OTF)_3_-H_2_O (1 M, 3 M) were used as electrolyte to tranform the spinel Mn_3_O_4_. As shown in Supplementary Figrue 6 a, neither of the sample displays the layered structure with d spacing of 4.94 Å, which is observed as a peak at 18^o^ in XRD pattern of the sample foromed in Al(OTF)_3_-H_2_O (5 M). Furthermore, the morphology of samples formed in Al(OTF)_3_-H_2_O (1 M, 3 M) are different from the nanopartiles of Al_x_MnO_2_·*n*H_2_O (Supplementary Figure 5d). This phenomenon indicates the Al_x_MnO_2_·*n*H_2_O is only formed in higly concentrated aqueous electrolyte (Al(OTF)_3_-H_2_O (5 M) electrolyte).

### Supplementary Notes 3: Confirmation of anode reaction.

To investigate whether the Al is result from the anode or electrolyte, the Al foil was weighted before and after discharge to varied depth in Al/Al(OTF)_3_-H_2_O/Al_x_MnO_2·_*n*H_2_O cells. If the capacity is resulted from Al stripping which is the electrochemical dissolving of Al foil, the theoretical anode weight loss is calculated according to the Faraday’s First Law of Electrolysis:

m=M·Q/n·F

=(m_(cathode materials)_·n_(cathode materials)_·M_(Al)_)/(M_(cathode materials)_·n_(Al)_)

=3.6(C_(cathode materials)_·m_(cathode materials)_M_(Al)_)/(n_(Al)_·F).

The cathode materials are Mn_3_O_4_ on which base the specific capacity were calculated, the n(Al)=3, M(Al)= 27 g mol^−1^. The experimental and theoretically weight loss of Al are listed in Supplementary Table 3. The weight loss of Al foil are close to the theoretical values, implying the striping of Al dominate anode reaction in discharge. The causes of higher experimental weight loss than theoretical value may be the chemical reactions that removing the passive oxidation layers on the Al foil^10^ and leading to weight loss. The striping of Al on anode is further confirmed by the pits and dots on the Al anode (Supplementary Figure 8).

## Supplementary References

1. Li, Z., Xiang, K., Xing, W., Carter, W.C. & Chiang, Y. Reversible aluminum-ion intercalation in Prussian blue analogs and demonstration of a high-power aluminum-Ion asymmetric capacitor. *Adv. Energy Mater.* **5**, 1401410–1401416 (2015).

2. Liu, S. et al. Aluminum storage behavior of anatase TiO_2_ nanotube arrays in aqueous solution for aluminum ion batteries. *Energy Environ. Sci.* **5**, 9743–9746 (2012).

3. Yu, X. & Manthiram, A. Electrochemical energy storage with a reversible nonaqueous room-temperature aluminum-sulfur chemistry. *Adv. Energy Mater.* **7**, 1700561–1700570 (2017).

4. Mori, T. et al. Discharge/charge reaction mechanisms of FeS_2_ cathode material for aluminum rechargeable batteries at 55°C. *J. Power Sources* **313**, 9–14 (2016).

5. Wang, S. et al. High-performance aluminum-ion battery with CuS@C microsphere composite cathode. *ACS Nano* **11**, 469–477 (2017).

6. Hu, Y. et al. An innovative freeze-dried reduced grapheneoxide supported SnS_2_ cathode active material for aluminum-ion batteries. *Adv. Mater.* **29**, 1606132–1606137 (2017).

7. Wang, S. et al. A novel aluminum-ion battery: Al/AlCl_3_-[EMIm]Cl/Ni_3_S_2_@Graphene. *Adv. Energy Mater.* **6**, 1600137–1600147 (2016).

8. Jayaprakash, N., Das, S.K. & Archer, L.A. The rechargeable aluminum-ion battery. *Chem. Commun.* **47**, 12610–12612 (2011).

9. Suo, L. et al. "Water-in-salt" electrolyte enables high-voltage aqueous lithium-ion chemistries. *Science* **350**, 938–943 (2015).

10. Wang, H. et al. Anion-effects on electrochemical properties of ionic liquid electrolytes for rechargeable aluminum batteries. *J. Mater. Chem. A* **3**, 22677–22686 (2015).
